# Age effects on voluntary and automatic adjustments in anti-pointing tasks

**DOI:** 10.1007/s00221-015-4459-6

**Published:** 2015-10-26

**Authors:** Marion Verneau, John van der Kamp, Michiel P. de Looze, Geert J. P. Savelsbergh

**Affiliations:** Research Institute Move, Faculty of Behavioral and Movement Sciences, VU University Amsterdam, B-656, van der Boechorststraat 7, 1081 BT Amsterdam, The Netherlands; Institute of Human Performance, University of Hong Kong, Pokfulam, Hong Kong; Research Institute for Biomedical Research into Human Movement and Health, Faculty of Science and Engineering, Manchester Metropolitan University, Manchester, UK; TNO, Quality of Life, Hoofddorp, The Netherlands

**Keywords:** Aging, Goal-directed, Stimulus-driven, Anti-pointing

## Abstract

We examined the effects of age on automatic and voluntary motor adjustments in pointing tasks. To this end, young (20–25 years) and middle-aged adults (48–62 years) were instructed to point at a target that could unexpectedly change its location (to the left or right) or its color (to green or red) during the movement. In the location change conditions, participants were asked to either adjust their pointing movement toward the new location (i.e., normal pointing) or in the opposite direction (i.e., anti-pointing). In the color change conditions, participants were instructed to adjust their movement to the left or right depending on the change in color. The results showed that in a large proportion of the anti-pointing trials, participants made two adjustments: an early initial automatic adjustment in the direction of the target shift followed by a late voluntary adjustment toward the opposite direction. It was found that the late voluntary adjustments were delayed for the middle-aged participants relative to the young participants. There were no age differences for the fast automatic adjustment in normal pointing, but the early adjustment in anti-pointing tended to be later in the middle-aged adults. Finally, the difference in the onset of early and late adjustments in anti-pointing adjustments was greater among the middle-aged adults. Hence, this study is the first to show that aging slows down voluntary goal-directed movement control processes to greater extent than the automatic stimulus-driven processes.

## Introduction

Typically, middle and late adulthood are accompanied by a decline in motor performance and learning. Middle-aged and old adults’ motor performance is slower and less fluent than the performance of young adults. Similarly, motor learning tends to slow down and becomes more effortful with age (Bleecker et al. 1985; Salthouse 1990; Salthouse and Somberg 1982; Schwerha et al. 2007; Shea and Kohl 1990; Yan 2000). Interestingly, however, the decline in motor learning especially arises when learning is explicit or deliberate, whereas implicit motor learning remains relatively spared in middle or late adulthood (Chauvel et al. 2011, 2012; Howard and Howard 2001; Verneau et al. 2014). Based on the assumption that motor learning growths directly out of movement control processes (Willingham 1998; Willingham and Goedert-Eschmann 1999), Verneau et al. (2014), hypothesized that the decline in explicit learning might reflect an isolated slowing down of explicit or deliberate control processes, which does not extend to the implicit or automatic movement control processes. Indeed, Verneau et al. (2014) reported that for a sequential motor task, in which participants had to respond to an ordered sequence of events, the age-related decline in the use of explicit processes was modulated by time constraints: The fewer time available between the events, the more difficulty older adults had to adequately use explicit instructions to produce the movement responses, resulting in poor performance and learning. By contrast, implicit learning was retained even under the most severe time constraints. This suggests that explicit control processes that operate on a longer timescale are more adversely affected by age than the fast control processes. The current study seeks to find further evidence that the slower deliberate or explicit control processes that support voluntary movement responses and the faster implicit control processes that underlie the more automatic movement responses are indeed differentially affected by age.

The processes underlying slow and fast movement responses have often been investigated in rapid aiming tasks. For example, participants are instructed to make fast pointing movements to a target displayed on a monitor in front of them, but to interrupt the movement when the target (abruptly) shifts to another location. Stopping, however, costs time. Hence, if participants indeed move fast, they often have difficulties deliberately stopping the pointing movement before they reach the target. Yet, it has been shown that, even if they fail to stop the pointing movement, they unwillingly and automatically make fast adjustments (i.e., within 200 ms after target shift) toward the new target location (Cressman et al. 2006; Pisella et al. 1998, 2000). However, if participants move slower, they can halt the movement. This indicates that two control processes are involved in adjusting ongoing movements: a goal-directed process that is slow, explicit and allows for voluntary movement adjustments and a stimulus-driven process that is fast, implicit and permits automatic movement adjustments (Corbetta and Shulman 2002; Day and Lyon 1994, 2000; Pisella et al. 1998). Similarly, in anti-pointing tasks, participants are either instructed to follow the target when it shifts location or to point toward the side opposite of the target shift (Day and Lyon 2000; see also Cressman et al. 2010). In the latter anti-pointing condition, participants typically first produce a fast adjustment in the direction of the new target location, which they did not intend to make, followed by an adjustment to the opposite side. Again, this is taken to reflect the workings of a fast automatic stimulus-driven and a slow deliberate goal-directed process, respectively. Here, the goal-directed process not only has to inhibit the ongoing movement (as in the stop task above), but also has to modify the pointing movement in accordance with the instruction.

The anti-pointing task is thus a tool par excellence for studying the operation of slow and fast movement control processes, but thus far has barely been used in older adults. Nonetheless, Rossit and Harvey (2008) instructed young and older adults to either adjust or stop their ongoing pointing in response to a target location shift. Older adults took longer than the young participants to adjust and inhibit (i.e., stop) their pointing movement. Accuracy, however, was not negatively affected by age (Potter and Grealy 2008; cf. Sarlegna 2006). Moreover, this slowing down was not more pronounced than when reaching for non-moving, stationary targets. Hence, Rossit and Harvey (2008) argued that their findings indicate a general slowing down, rather than specific decline of either the slow goal-directed or fast stimulus-driven process (albeit that they did not compare the two processes directly). Importantly, however, the stop task only requires a deliberate inhibition of the ongoing pointing movement, not a voluntary re-adjustment toward a new location as is required in the anti-pointing task. Thus far, middle-aged and older adults’ performance on the anti-pointing task has not been studied. Hence, we examined and compared the fast automatic and slow voluntary adjustments in an anti-pointing task among young and middle-aged adults.

Yet, although the anti-pointing task potentially allows disentangling simultaneous contributions of the slow goal-directed and fast stimulus-driven processes, it cannot control for their interaction. For example, the slowing down of the voluntary re-adjustment of the pointing movement may or may not be enhanced by the need to override the fast automatic adjustment. Hence, to ensure that any observed age-related slowing down truly reflects the goal-directed process, we also examined a variant of the target displacement paradigm that does not trigger automatic adjustments. In this variant, the requirement to stop or redirect the ongoing pointing is signaled by a change in target color (Veerman et al. 2008). Contrary to a shift in the spatial location of the target, a color change does not set off a fast automatic adjustment (Cressman et al. 2006; cf. Brenner and Smeets 2004). Consequently, adjustment to color change is purely voluntary, and—presumably—uniquely relying on the goal-directed process. A direct comparison between the adjustments of anti-pointing in response to a location shift and a color change thus allows to disentangle whether the anticipated age-related differences merely reflect the slowing down of the goal-directed process or also its interaction with the automatic stimulus-driven process.

In sum, we compared middle-aged and young adults’ pointing performance in response to a target that shifts its location or changes color. We expected the explicit goal-directed process to slow down more (i.e., to take more extra time) with age than the automatic stimulus-driven process. Hence, it was hypothesized that the latency of the voluntary redirection of the anti-pointing movements would be more adversely affected by age than the initial fast automatic movement adjustments. It is hypothesized that if this slowing down purely reflects an age-related decline of the goal-directed process, then it would be of similar magnitude for a spatial target shift than for a target color change; however, if the slowing down (also) reflects the interaction with the stimulus-driven process, then the voluntary adjustment should be slower in response to the spatial target shift than to a color change.

## Methods

### Participants

Nine young (three women, *M* = 23.6 years, SD = 1.8) and nine middle-aged (four women, *M* = 55.7 years, SD = 3.8) adults volunteered to participate in the experiment. All participants were self-proclaimed right-handers, had normal or corrected-to-normal vision, and were healthy with no history of neurological or movement disorders. None of them was color-blind. Local ethics committee approved the protocol, and all participants gave their informed consent prior to the experiment.

### Apparatus and stimuli

Participants were seated in front of a table, on which a vertically oriented LCD computer screen (38 × 30 cm) stood. They sat in a chair positioned in such a way that they could comfortably contact the screen with their right index finger. Floor marking was used to make sure that the table and the chair positions were kept constant during the entire experiment. On the table, a command button was fixed in front of the participants at 27.5 cm from the screen and 1 cm to the left of the middle of the screen.

**Fig. 1:**
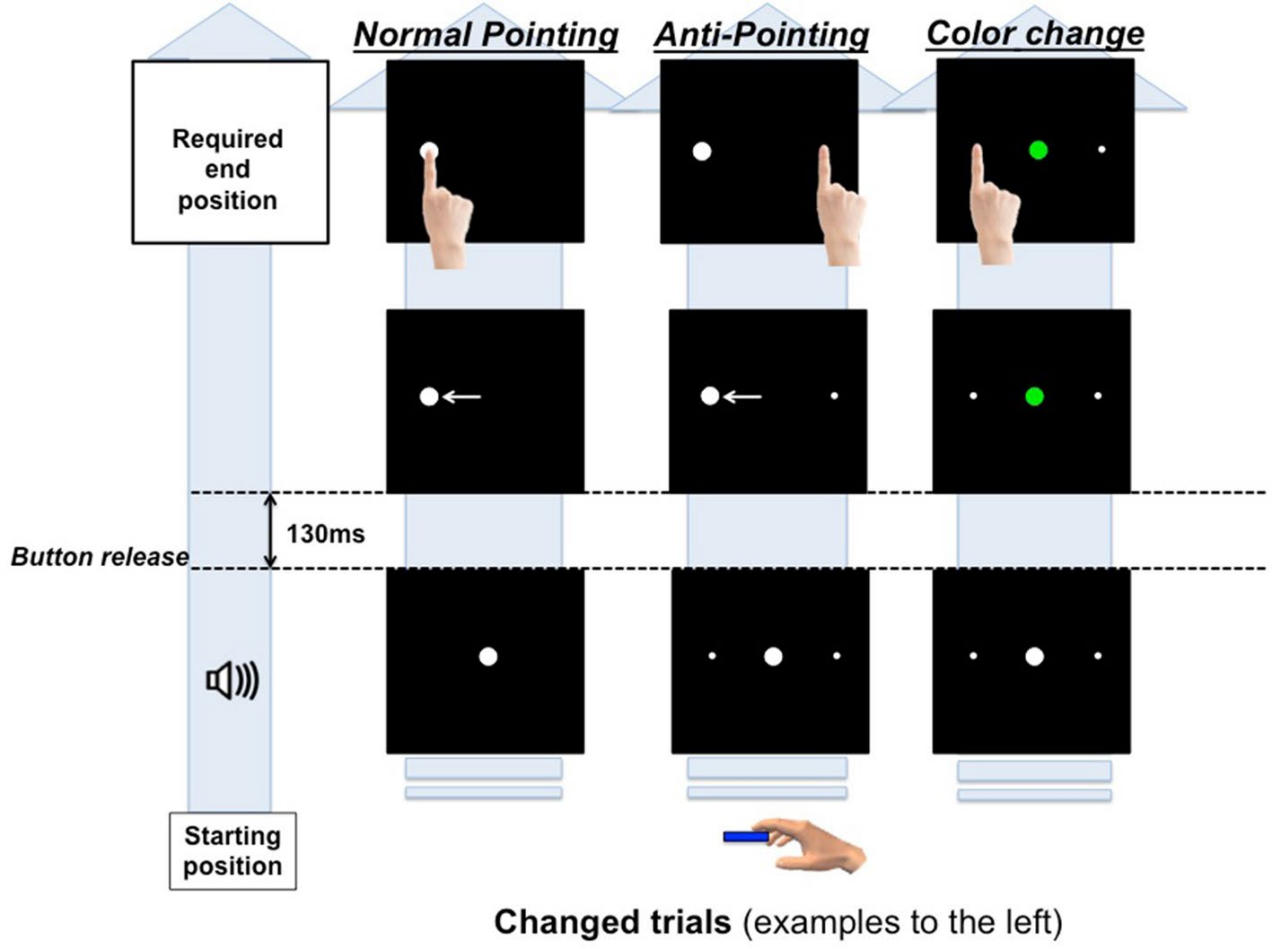
From *left* to *right*, the change-trials are illustrated for the normal pointing, anti-pointing and color change conditions. For each condition, the trial proceeds from *bottom* to *top*

E-prime2 (Psychology Software Tools) was used to run the experiment. The stimulus displayed was a white circle of 2.0 cm diameter (i.e., the target) on a black background. The initial position of the circle was in the middle of the screen, 30 cm above the table. When the target changed location, its position jumped laterally either 7 cm to the right or 7 cm to left from the initial position. During the color change, the initial white target switched to red or green color. During the anti-pointing and color change conditions, two small dots (i.e., 0.05 cm ø) were always present 7 cm on each side of the initial target location (see Fig. 1). These non-salient small dots were used to reduce spatial uncertainty during these two conditions (i.e., indicated the pointing location).

An Optotrak Certus Motion Capture System (Northern Digital Inc.) recorded the 3D position of 5 IREDs at 500 Hz. The Optotrak cameras were positioned 2 m to the left of the participants at an approximate height of 2 m. Two IREDs were positioned on the right index finger (i.e., on the left side of the nail and the left lateral side above the border of the proximal and middle phalanxes). The three remaining IREDs were placed in the action space: one at the start button and two at the bottom left and top right corner of the computer screen. The start button was where the index finger rested at the beginning of each trial (i.e., starting position). That is, before each trial, the participants pressed on the start button (and IRED) with their right index finger. The starting button was released the moment the participant started reaching. This generated a signal, which was fed into the Optotrak and the E-prime systems. The latter then triggered the location shift or color change in the target after a 130-ms delay (see e.g., Komilis et al. 1993).

Finally, the Digital Memory Span test from the Wechsler Adult Intelligence Scale III (WAIS-iii) was used to assess working memory functioning.^1^ This test involved remembering an increasingly larger set of numbers in a specific sequence. Furthermore, participants were required to fill in a questionnaire with general questions regarding neurological, movement and visual impairments (including color blindness) or disorders, medication use and handedness.

### Design

The experiment consisted of one session that took approximately 75 min during which participants performed pointing tasks in three conditions: normal pointing, anti-pointing and color change conditions. However, participants first performed the digit span test and were then provided with instructions about the procedure and tasks. Subsequently, the IREDs were positioned on the index finger, and a final calibration procedure was conducted (i.e., consecutive pointing movements to the three target locations).

The order of the normal pointing, anti-pointing and color change conditions was counterbalanced across participants in each group. For each condition, a total of 120 trials were completed, in which the target could change (i.e., in location or color) or not. A target change required the participants to adjust the direction to which they were pointing; these trials were labeled as ‘change’-trials. In the remaining trials, the target neither shifted location nor changed color, and no adjustment in the direction of pointing was required; these trials were dubbed ‘non-change’-trials. Each trial started with the index finger pressing the starting button. Then a white target appeared at the center of the screen. Participants waited (for approximately 2 s) until a short auditory cue signaled that they could start the pointing movement. They were instructed to move their index finger toward the target as fast and accurately as possible. In addition, they were instructed to keep the finger on the screen until a text appeared that told them to return the index finger to the starting position. In the normal pointing condition, there were 80 non-change-trials and 40 change-trials, during which the target shifted to the right (20 trials) or to the left (20 trials) 130 ms after the start button was released. For the change-trials, participants were instructed to adjust pointing direction to the new target location. To avoid anticipation of change-trials, the 120 non-change- and change-trials were presented in random order. In the anti-pointing condition, the 120 trials were organized into non-change- and change-trials as in the normal pointing condition. Yet, in change-trials, participants were required to adjust pointing direction to side (i.e., the small dot) opposite of the side to which the target shifted. Finally in the color change condition, the target did not shift position. However, in the 40 change-trials, the target changed from white to green (20 trials) or to red (20 trials) 130 ms after the start button was released. In the 80 non-change-trials, the target remained white. In the case the target changed to red, participants were instructed to adjust pointing direction to (the small dot on) the right; if the target turned green, they were to adjust pointing direction to the left. Figure 1 summarizes for the procedure for the change-trials in the three conditions.

### Data analysis

To start with, we identified all trials during which participants started pointing before the auditory signal. These trials were excluded from analyses (i.e., 1 %). We subsequently identified the change-trials during which the participants pointed to the wrong side (i.e., faulty trials), determined the descriptive kinematics of the pointing movements (i.e., reaction time, movement time, endpoint accuracy and endpoint precision), and finally, determined the time participants needed to adjust pointing direction in response to the target change. We discuss the analyses in this order.

#### Identifying faulty trials

To identify whether or not the pointing movement in change-trials ended at the incorrect side (e.g., during anti-pointing movements ending at the left side for a target shift to left side or in normal pointing at the left side for a target shift to right), we evaluated the spatial trajectory of the index finger. The pointing movement was classified as faulty if the finger approached the wrong target within a sphere with a radius of 30 mm for a minimum duration of 150 ms. For each condition, the percentage of trials in which a faulty pointing movement was made was calculated. The faulty trials were removed for further analyses.

#### Descriptive kinematics

For each pointing movement, reaction time, movement time, endpoint accuracy and endpoint precision were determined. The reaction time was calculated by taking the time difference between the onset of the auditory signal and the onset of the button release. The movement time was defined by taking the time between the end of the pointing movement and the button release. The end of the pointing movement was defined as the moment the index finger touched the screen. From this, the endpoint accuracy was determined by calculating the average distance between the required end location (i.e., the location of the ‘target’) and the actual location of the index finger at the end of the movement, while the standard deviation of the average distance between the required end location and actual endpoint of the index finger gave the endpoint precision.

#### Identifying the onset of the adjustments

Following previous work, the evolution of the lateral pointing speed of non-change-trials and change-trials was compared to detect the onset of adjustments in the pointing movements in response to the target change (e.g., Cressman et al. 2010; Day and Lyon 2000). For both the normal pointing and color change conditions, this was done by comparing all speed profiles for the non-change-trials to all speed profiles of the change-trials (i.e., target shifts to left and right separately). That is, for each participant and condition, the differences in lateral speed between the two sets of profiles were compared for each sample from the onset of target change until 1200 ms thereafter by using *t* tests.^2^ The first sample at which the sets of lateral speed were significantly different was taken as the onset of the adjustment, with the moment of onset being defined relative to the moment of target change (i.e., location shift and color change).

**Fig. 2:**
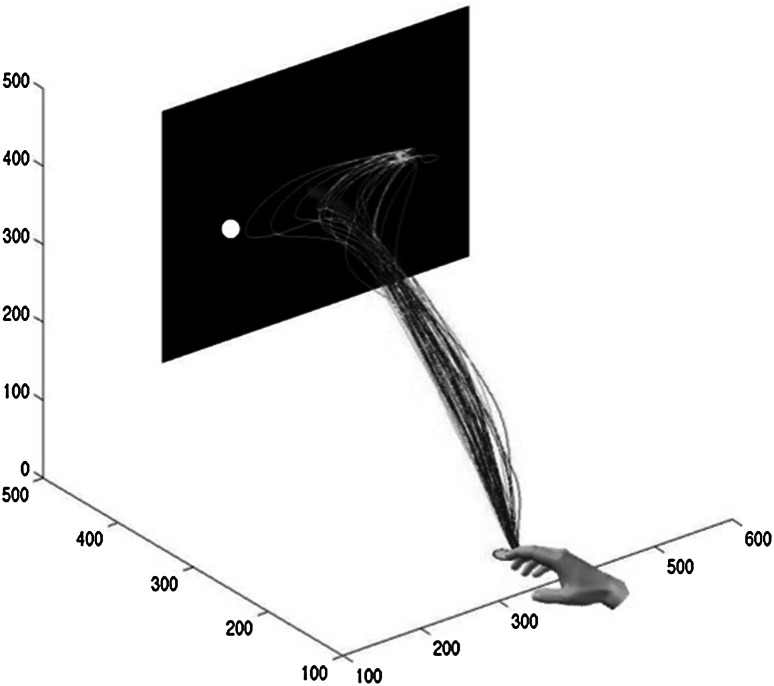
Three-dimensional representation of non-change (*blue lines heading toward the center of the screen*) and change-trials to the left (*green lines finishing on the right side of the screen*) in the anti-pointing condition for a young participant. The *black rectangle* represents the screen, and the green circle represents the starting button

**Fig. 3:**
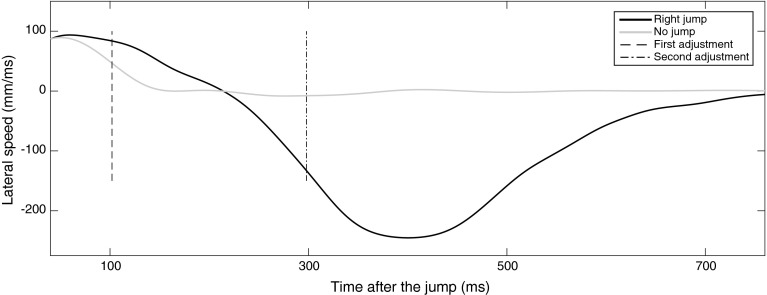
Averaged change-trial (*black*) and non-change-trial (*grey*) lateral speed profiles in the anti-pointing condition for a young participant. Indicated are the first or early adjustment (--) and the second or late (-.-) adjustment to a target shift to the right (at 102 and 298 ms, respectively)

In the anti-pointing condition, often (but not always) two adjustments were made. As depicted in Figs. 2 and 3, after the target change (e.g., to the right), often a first adjustment in direction of the target shift (i.e., to the right) occurred, followed by a second adjustment toward the correct side (i.e., the anti-point to the left). In other trials, however, only the second or late (anti-point) adjustment was observed. Hence, a trial-by-trial analysis was necessary to sort out trials with two adjustments from those with only one adjustment. To do so, we compared the lateral speed profile of each individual change-trial to the average lateral speed profile of all the non-change-trials. This comparison was based on a speed difference threshold. This threshold was defined as the minimum divergence between the speed profiles of the non-change-trials and change-trials that can be considered reliably different. Hence, we performed a series of *t* tests between the sets of non-change-trials and change-trials to find the first sample at which the two sets of speed profiles were significantly different. The corresponding speed difference between two sets of speed profiles served as the speed difference threshold. Subsequently, for each individual change-trial, the first or early adjustment was identified if 80 ms (i.e., the estimated minimum visuo-motor delay) or longer after target shift, the difference between lateral speed for that individual change-trial profile and the average lateral speed profile of all the non-change-trials exceeded the speed difference threshold for at least 70 ms (see Cressman et al. 2010) and if the adjustment was indeed directed to the incorrect side (i.e., by visual inspection and as reflected by the sign of the speed difference). The percentage of change-trials with two adjustments was calculated.

Next, the moment of onset of the first or early adjustment in the anti-pointing trials with two adjustments was determined in the same way as the onset of adjustment in the normal pointing and color change conditions (see above). That is, using a series of *t* tests, the onset was defined as the sample at which the set of lateral speed profiles of the change-trials (with two adjustments) differed significantly from the set of speed profiles of the non-change-trials. The moment of onset was then defined relative to the moment of target change. Finally, the second or late adjustment was calculated for trials with one and two adjustments separately. It was defined as the sample at which the set of lateral speeds of the non-change-trials and change-trial were significantly different and the change-trial lateral speed was directed toward the *correct* side (e.g., negative lateral speeds for anti-pointing to the right). The moment of onset was then defined relative to the moment of target change.

Outliers in time of adjustment were detected following the outlier labeling method of Hoaglin (Hoaglin and Iglewicz 1987; Hoaglin et al. 1986), in which outliers are defined relative to lower and upper bounds for percentiles scores (i.e., lower bound = P25 − [(P75 − P25) × 2.2] and upper bound = P75 + [(P75 − P25) × 2.2]).

Finally, to assess a putative different impact of age on fast automatic and slow goal-directed adjustments, the difference between the moments of the first or early and second or late adjustments was also calculated for each age group.

### Statistics

First, to assess participants’ ability to respond adequately to target changes (i.e., point to the instructed side), the percentage of faulty trials was submitted to a 2 (age: young adults, middle-aged adults) by 3 (condition: normal pointing, anti-pointing, color change) ANOVA with repeated measures on the last factor. Similar ANOVA’s with repeated measures were conducted to examine differences in reaction time, movement time, endpoint accuracy and endpoint precision as function of age and condition. Next, an independent two-tailed *t* test was used to compare the percentage of anti-pointing change-trials with two adjustments between young and older adults. Most important, however, were the comparisons for the moments of adjustment. First, a 2 (age: young adults, middle-aged adults) by 4 (adjustment: normal pointing, early adjustment anti-pointing, late adjustment anti-pointing, color change) ANOVA with repeated measure on the last factor was performed over the moment of adjustment. Second, a two-tailed independent *t* test was used to assess whether the difference between the moments of the first or early and second or late adjustments differed between the two age groups. For all the above analyses, post hoc comparisons were performed using *t* tests with Bonferroni corrections (α = 0.05). Partial eta-square (ηp^2^) and Cohen’s d were reported to determine the proportion of total variability attributable to each factor or combination of factors.

## Results

### Percentage of faulty pointing movements

There were only five trials (out of a total of 2160 change-trials) that terminated at the wrong side, which occurred exclusively among the young participants. Not surprisingly, therefore, the ANOVA did not reveal any significant effects for the percentage of faulty movements. The faulty trials were excluded from further analyses.

### Descriptive kinematics

Table 1 reports the reaction time, movement time, endpoint accuracy and endpoint precision as a function of age and condition. The ANOVAs revealed significant main effects of condition for movement duration [*F*(2, 32) = 156.3, *p* < 0.001, ηp^2^ = 0.91] and endpoint precision [*F*(2, 32) = 11.5, *p* < 0.001, ηp^2^ = 0.42]. However, for the endpoint accuracy, the condition effect almost reached significance [*F*(2, 32) = 3.19, *p* = 0.052, ηp^2^ = 0.17]. Yet, neither the main effects of age nor the interactions between age and condition were significant. The post hoc analyses indicated that the movement duration was smallest in the normal pointing condition and longest in the color change condition. For the endpoint position, participants (in both groups) were less precise and tended to be less accurate in the anti-pointing condition compared to the other conditions.

**Table 1:** Means (and standard deviations) for reaction time, movement time, endpoint accuracy and endpoint precision as a function of age and condition

	Reaction time (ms)		Movement duration (ms)		Endpoint accuracy (mm)		Endpoint precision (mm)	
	Young	Middle-age	Young	Middle-age	Young	Middle-age	Young	Middle-age
Pointing	160 (40)	193 (42)	632 (144)	672 (107)	9 (2)	7 (3)	5 (2)	4 (3)
Anti-pointing	162 (53)	204 (40)	686 (79)	774 (126)	10 (3)	7 (3)	7 (3)	5 (3)
Color	163 (41)	204 (38)	745 (104)	836 (124)	9 (3)	6 (2)	5 (3)	4 (2)

### Percentage of anti-pointing trials with two adjustments

Not all participants showed two adjustments (i.e., a first or early and a second and late adjustment) in response to the target shift in all the change-trials of the anti-pointing condition. The *t* test for the percentage of anti-pointing change-trials with two adjustments revealed a significant difference between the two age groups [*t*(16) = 2.9, *p* < 0.05, *d* = 1.4], indicating that the younger adults less often showed two adjustments (i.e., *M* = 45.0 %, SD = 12.1) than the middle-aged participants (*M* = 65.8 %, SD = 17.5).

### Moments of onset adjustment

**Fig. 4:**
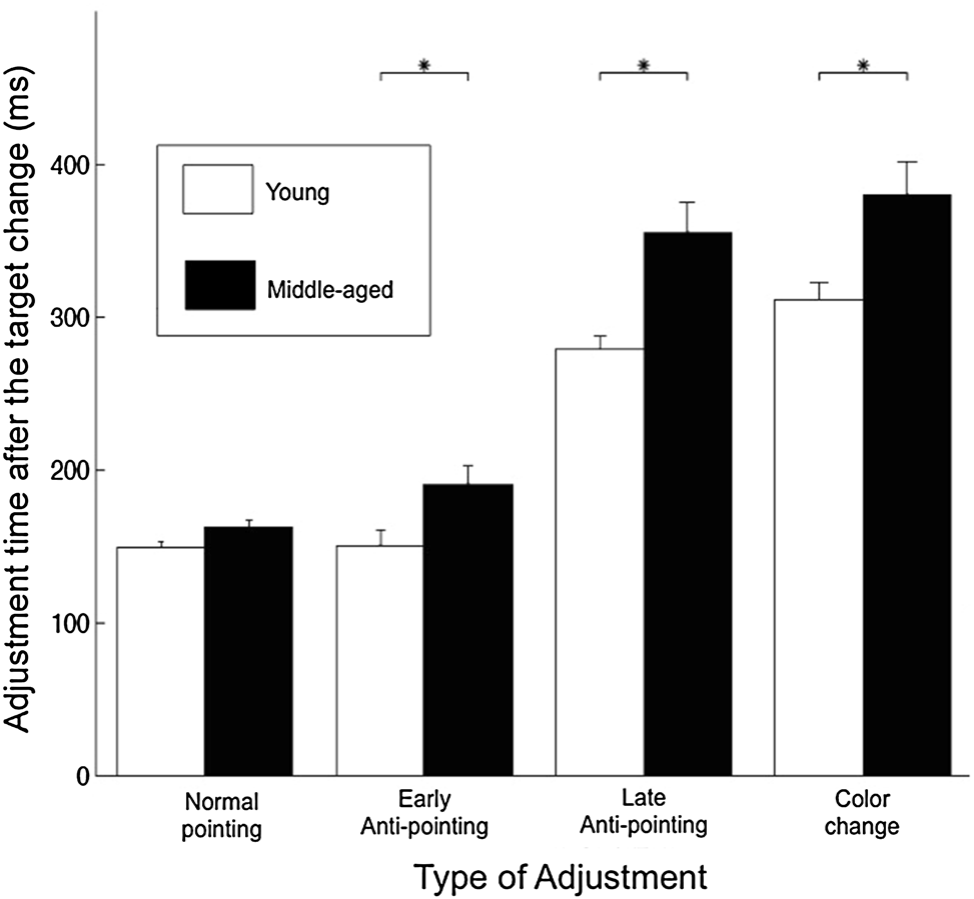
Moments of adjustment during change-trials for young and middle-aged adults in normal pointing, anti-pointing (i.e., first or early and second or late adjustment) and color change conditions

One of the young adults was identified as an outlier for the moment of the late adjustment in the anti-pointing condition and was removed from subsequent analysis.^3^ Figure 4 shows the moments of adjustments. It suggests that the middle-aged participants tended to react slower to the target change than the young adults. This age difference, however, appears less pronounced in the normal pointing task than in the anti-pointing and the color change task. Accordingly, the ANOVA revealed main effects of age [*F*(1, 15) = 10.95, *p* < 0.01, ηp^2^ = 0.42] and adjustment [*F*(3, 45) = 206.01, *p* < 0.001, ηp^2^ = 0.93] as well as a significant interaction between the two factors [*F*(3, 45) = 4.52, *p* < 0.05, ηp^2^ = 0.23]. Post hoc analysis confirmed that with the exception for the adjustment in the normal pointing condition, the middle-aged adults initiated the adjustments later than the younger adults. It also indicated that for both age groups, neither the adjustment times for the first or early adjustments (i.e., normal pointing and the first or early adjustment in anti-pointing) nor the adjustment times for the second or late response (i.e., color change and the second or late adjustment in anti-pointing) were different. Finally, a *t* test showed that the difference between the moments of the first or early and second or late adjustments under anti-pointing condition significantly differed between the two age groups [*t*(15) = 2.8, *p* < 0.05, *d* = 1.4]. The difference was greater among the middle-aged participants (i.e., *M* = 164 ms, SD = 27) than for the younger participants (*M* = 128 ms, SD = 26) (see also Fig. 4).

## Discussion

In the current experiment, we used various pointing tasks to investigate the effects of age on automatic stimulus-driven and deliberate goal-directed movement control processes. Young and middle-aged participant pointed to targets that either shifted location, changed color or, in the majority of trials, remained unchanged. The required adjustments in response to the target change were either in the same or in the opposite direction as the target change. In particular, the anti-pointing task allowed for a direct comparison between the two control processes, with a fast early adjustment in the direction of the target shift reflecting the automatic stimulus-driven process and a slower second adjustment reflecting the deliberate goal-directed process. The present results indicate that the effects of aging are most pronounced for the second adjustment, suggesting that the slowing down is more distinct for the goal-directed process than for stimulus-driven process. However, before elaborating this conclusion, we first discuss the findings for the two processes separately.

### The automatic stimulus-driven process

In approximately half of the anti-pointing trials, the participants showed an early adjustment in the pointing trajectory in the same direction as the target shift. Since, participants were instructed to point to the side opposite to which the target shifted, we can conclude that this early adjustment is involuntary or automatic. Consequently, it reflects the operation of the fast automatic stimulus-driven process. The early anti-pointing adjustment was observed in both age groups and occurred at roughly the same time after the target change than during the normal pointing task (i.e., approximately 170 and 155 ms, respectively). The equivalent fast scale at which these two adjustments occurred indicates that spatial adjustments during normal pointing were under control of the same stimulus-driven process as the initial automatic adjustment in the anti-pointing trials.

The early adjustment in the anti-pointing task was somewhat slower for middle-aged adults than for young participants, whereas no age difference arose for the fast adjustment in the normal pointing task. This might suggest a slowing down with aging of the automatic stimulus-driven process, but only when it operates in the context of anti-pointing. Yet, this interpretation is not fully supported by the present findings: The moment of the adjustments in the normal pointing and the moment of the first adjustment in the anti-point trials did not significantly differ among the middle-aged adults (nor did they differ among the young adults). Further research is needed to resolve this ambiguity (e.g., by assessing the impact of additional visual stimuli) (Cameron et al. 2007; Hasher et al. 1999) and to determine whether there is a genuine age-related slowing down of the automatic processes during the anti-pointing task. At this stage, we conclude that there is no unequivocal evidence that the automatic stimulus-driven process appreciably slows down before 60 years of age.

### Voluntary adjustment

As anticipated, the late or second adjustment in the anti-pointing task toward the opposite side of the target shift was delayed relative to the adjustment during normal pointing in the direction of the target shift. Importantly, the late adjustment in the anti-point task was noticeably slower for middle-aged than for young participants. The same was true for the adjustment in response to the color change. In fact, for both age groups, these voluntary adjustments in response to the color change occurred at the same moment as the anti-pointing in response to the target shift. Consequently, the slowing down of the late anti-pointing adjustment observed in middle-aged adults purely reflects the slowing down of the voluntary goal-directed process untainted by interactive influence of the early adjustment (e.g., the inhibition of the initial fast adjustment). Crucially, the absolute difference in the response times for the initial fast and the second slow adjustments was greater for the middle-aged adults than for the younger adults.^4^ Hence, this shows that not only the goal-directed movement control process is slowed down by aging, but additionally that this age-related slowing down is clearly more pronounced than for the stimulus-driven process. It is, however, important to acknowledge that when the slowing down is expressed in percentages (i.e., by calculating the difference in adjustment times for young and middle-age adults divided by adjustment time for the young adults), then the age-related slowing downs for the stimulus-driven (i.e., 26 %) and goal-directed processes (i.e., 30 %) are of similar magnitude. Nonetheless, this does not alter the fact that in terms of behavioral consequences (i.e., the change in absolute increase in movement duration), the slowing down is much pronounced and meaningful for the goal-directed process than for stimulus-driven process.

An intriguing finding is that in the anti-pointing task, participants were capable of overriding or suppressing the initial fast adjustment, the proportion in which this happened being larger in young than middle-aged adults (i.e., 55 and 34 %, respectively). A voluntary modification of an automatic response appears contradictory, yet others reported similar observations in studies with young adults (Cameron et al. 2009; Day and Lyon 2000; McIntosh et al. 2010; Striemer et al. 2010). These observations show that the voluntary goal-directed and automatic stimulus-driven processes do interact. One might argue that participants who do expect a target shift, may in the course of an experiment become aware of the initial incorrect adjustments and try to repress them. Yet, additional analysis showed that participants did not repress the early fast adjustment more often during the second part of the anti-pointing condition than they did at the beginning.^5^ Perhaps, the slowing down of the goal-directed process with age weakens this interaction, and hence, middle-aged adults become less efficacious than younger adults in repressing the automatic process. McIntosh et al. (2010) argued that repressing the automatic adjustment is cognitively demanding (i.e., active inhibition). Although the cognitive cost was reduced when normal and anti-pointing trials were presented in separate blocks, the additional cognitive demands may be another reason why the middle-aged participants were less capable in suppressing the automatic response.

## Conclusion

The current results are the first that directly disclose that in terms of movement duration the voluntary goal-directed process slows down with age more than the automatic stimulus-driven movement control processes. This age-related slowing down may occur as early as 50 years of age. This slowing down appears specific to making adjustments to ongoing movements, rather than merely reflecting a difference in speed–accuracy trade-off (i.e., the middle-aged participants showed similar patterns of reaction time, movement time, endpoint accuracy and precision than the young adults). The present findings thus highlight the increased time that voluntary movement control processes consume among middle-aged adults; these voluntary processes are not necessarily becoming less accurate than automatic control movements, although prolonged movement duration may increase the chance of error. These findings are consistent with the observation for sequential motor learning demonstrating that age-related deterioration of explicit learning, supposedly involving the goal-directed process, can be alleviated by reducing the time constraints (Verneau et al. 2014). The time needed to manipulate and use explicit information seems a key factor in older adults’ greater loss of efficiency in explicit compared to implicit motor learning (Howard and Howard 2001; Verneau et al. 2014). Hence, both for motor performance and learning, it is pertinent to provide middle-aged and older adults sufficient time, at least when explicit instruction and voluntary control are important factors for task fulfillment.

## Footnotes

1 In fact, an independent two-tailed *t* tests did not reveal significant differences in memory span between groups [*t*(16) = 0.8, *p* > 0.05], and hence, it was not included in any further analyses.

2 The synchronization between the starting button (measured by E-prime) and the Optotrak recordings proved not fully reliable. Hence, we used Optotrak recordings to determine movement onset. The average delay between the moments of movement onset and target change was used to scale (or synchronize) non-change- to the change-trials.

3 This participant was also less inclined than others to make the first or early response in the direction of the target in the anti-pointing condition (i.e., in only 25 % of the change-trials two adjustments were observed).

4 It can be argued that this difference relates to or is caused by the older participants making two adjustments in the anti-pointing trials more often. However, an additional analysis including only those anti-pointing trials in which two adjustments were made revealed a similar effect of age on the moment of onset of the second adjustment.

5 For this analysis, the anti-point trials were divided into two blocks (i.e., first half and second half of the trials) and the number of trials with an early adjustment was submitted to a 2 (age: young adults, middle-aged adults) by 2 (block: first, second) ANOVA with repeated measure on the last factor. This only revealed a significant age effect, confirming earlier analyses that middle-aged participants showed more trials with an initial incorrect adjustment. However, there were no main or interaction effects of block.
